# Inhibition of the Warburg effect with a natural compound reveals a novel measurement for determining the metastatic potential of breast cancers

**DOI:** 10.18632/oncotarget.2689

**Published:** 2015-01-09

**Authors:** Ritu Arora, David Schmitt, Balasubramanyam Karanam, Ming Tan, Clayton Yates, Windy Dean-Colomb

**Affiliations:** ^1^ Department of Oncologic Sciences, University of South Alabama Mitchell Cancer Institute, Mobile, AL 36604, USA; ^2^ Department of Biology and Center for Cancer Research, Tuskegee University, Tuskegee, AL 36088, USA; ^3^ Department of Oncologic Research, University Hospital and Clinics, Lafayette General Health, Lafayette, LA 70503, USA

**Keywords:** Warburg effect, Metabolism, Panepoxydone, LDH-A, LDH-B, Breast cancer

## Abstract

Metabolism is an important differentiating feature of cancer cells. Lactate dehydrogenases (LDH) A/B are metabolically important proteins and are involved in the critical step of inter-conversion of lactate to pyruvate. Panepoxydone (PP), a natural NF-kB inhibitor, significantly reduces the oxygen consumption and lactate production of MCF-7 and triple negative (MDA-MB-231, MDA-MB-468 and MDA-MB-453) breast cancer cells. We further observed that PP inhibited mitochondrial membrane potential and the ATP synthesis using flow cytometry. PP also up-regulated LDH-B and down-regulated LDH-A expression levels in all breast cancer cells to similar levels observed in HMEC cells. Over-expression of LDH-B in cancer cell lines leads to enhanced apoptosis, mitochondrial damage, and reduced cell migration. Analyzing the patient data set GDS4069 available on the GEO website, we observed 100% of non TNBC and 60% of TNBC patients had less LDH-B expression than LDH-A expression levels. Herein we report a new term called Glycolytic index, a novel method to calculate utilization of oxidative phosphorylation in breast cancer cells through measuring the ratio of the LDH-B to LDH-A. Furthermore, inhibitors of NF-kB could serve as a therapeutic agent for targeting metabolism and for the treatment of triple negative breast cancer.

## INTRODUCTION

Relative to normal cells, cancer cells are highly proliferative and thus require increased ATP to meet their metabolic demand. Cancer cells fulfill this requirement by depending on a faster mode of energy production, even in the presence of oxygen, a phenomenon commonly referred to as the Warburg effect [1, 2]. In the presence of oxygen, normal cells generate energy from glycolysis coupled with oxidative phosphorylation, an efficient process yielding approximately 38 molecules of ATP for each molecule of glucose consumed [3]. Glucose enters the cell and, after a series of enzymatic reactions, generates pyruvate. Pyruvate then enters the mitochondria, where it is metabolized to CO_2_ and water through the formation of ATP [4]. Under anaerobic conditions, pyruvate undergoes fermentation and is converted to lactate by a reaction catalyzed by lactate dehydrogenase (LDH). However, in cancer cells, pyruvate is preferentially converted into lactate even in the presence of oxygen [5, 6].

LDH is a tetrameric enzyme, containing 2 major subunits (A and B), encoded by 2 different genes, LDH-A and LDH-B [7, 8]. Lactate dehydrogenase A (LDH-A; also known as LDH-M and LDH-5), which is the predominant form in skeletal muscle, kinetically favors the conversion of pyruvate to lactate. The other isoform, lactate dehydrogenase B (LDH-B; also known as LDH-H and LDH-1) preferentially catalyzes the reverse reaction, in which lactate is converted back to pyruvate [9].

LDH-A is over-expressed in various tumor types, including breast cancer [10, 11]. Several LDH-A inhibitors are reported in the literature [12, 13]. Inhibition of LDH-A leads to a reduction in cellular transformation, delayed tumor initiation, and inhibition of growth of breast cancer xenografts [14, 15]. Further, in both ER-positive and -negative breast cancer cells, loss of LDH-A results in increased mitochondrial-induced apoptosis via production of reactive oxygen species [16]. Thus, LDH-A is involved in breast cancer tumorigenesis.

In contrast to LDH-A expression, LDH-B is highly expressed in non-malignant tissues relative to tumors [17]. In malignant tumors, LDH-B is silenced by promoter hypermethylation; this occurs at a high frequency in primary breast tumors (100%, 25/25) and in primary prostate tumors (45%, 14/31) [17, 18]. Although most reports indicate that LDH-B expression is decreased in breast tumors [17], integrative genomic analysis found that LDH-B expression is higher in the basal-like versus luminal subtype of cells in triple-negative breast cancers (TNBCs), and silencing of LDH-B expression in MDA-MB-231 cells decreases tumor growth [19]. While these findings appear contradictory, one caveat is that, in most of these studies, LDH-A and LDH-B were studied individually, thus hampering a broader view of their roles in cancer metabolism.

In this study we determined enhanced apoptosis, mitochondrial damage, and reduced migration of cancer cells in over-expressed LDH-B breast cancer cells. Herein, we report that the LDH-B/LDH-A ratio reflects the metabolic capacity of breast cancer cells. We propose a new measurement, the “*Glycolytic Index*,” which quantitates the ratio in cancer cells and demonstrate the value of this ratio as a biomarker of breast cancer aggressiveness. Further, this measurement can be utilized in predicting the metastatic behavior of breast cancers.

## RESULTS

### PP treatment modulates bioenergetics in human breast cancer cells

We recently reported that, in TNBC cells, PP, a natural NF-ĸB inhibitor, induces apoptosis and causes a reversal of the epithelial-mesenchymal transition [20]. We determined the effect of PP on breast cancer cells to form colonies. In the presences of PP, the number of colonies formed by breast cancer cells was significantly less than the control cells (Supplementary Figure 1). Since NF-κB regulates energy homeostasis via engagement of the cellular networks governing glycolysis and respiration [21], PP was utilized to investigate the role of NF-ĸB in cancer-related bioenergetics, a feature that differentiates cancer cells from normal cells [22].

**Figure 1:**
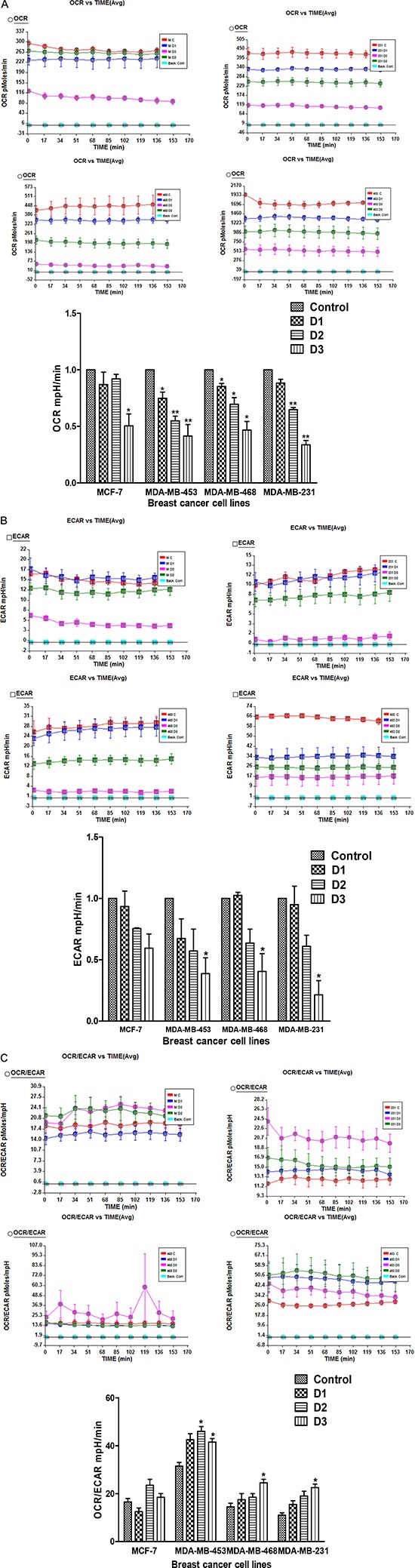
PP treatment inhibits oxidative phosphorylation (OXPHOS) in breast cancer cells **(A)** Effect of PP on oxygen consumption rate (OCR), an indicator of OXPHOS, in MCF-7, MDA-MB-231, MDA-MB-468 and MBA-MD-453 cells following 24 hr of treatment with DMSO (control) or the indicated concentrations of PP. *D1, D2, and D3s are half the IC_50_, the IC_50_, and double the IC_50_ concentrations. **(B)** Effect of PP on basal ECAR levels. **(C)** Effect of PP on OCR to ECAR ratio in breast cancer cells. Data shown are means ± SEM of two independent experiments, each performed in triplicate. Asterisks indicate statistically significant differences between PP-treated and untreated cells, *p* < 0.05 (*), and *p* < 0.01(**), as determined by Student's t-test.

The IC_50_ values of PP in different cell lines were 4uM, 5uM, 8uM and 15uM, respectively for MDA-MB-453, MCF-7, MDA-MB-468 and MDA-MB-231 cell lines. All these breast cancer cell lines were treated with concentrations of PP equal to- half the IC_50_, the IC_50_, and double the IC_50_ for 24 hr and were analyzed for their oxygen consumption rate (OCR), an indicator of oxidative phosphorylation (OXPHOS), and their extracellular acidification rate (ECAR), utilizing the Seahorse Analyzer. All breast cancer cell lines showed decreases in OCR after 24 hr of exposure to PP (Figure 1A). However, significant decreases in ECAR were noticed only in MDA-MB-231 and MDA-MB-453 cells (Figure 1B). Overall, the results suggest that PP reduces lactate production in breast cancer cells. The ratio OCR/ECAR, an indicator of oxidative phosphorylation, was increased in all the TNBC cells after PP treatment; this was not observed for MCF-7 cells (Figure 1C).

### PP induces apoptosis through reduced mitochondrial membrane potential (Δψm)

**Figure 2:**
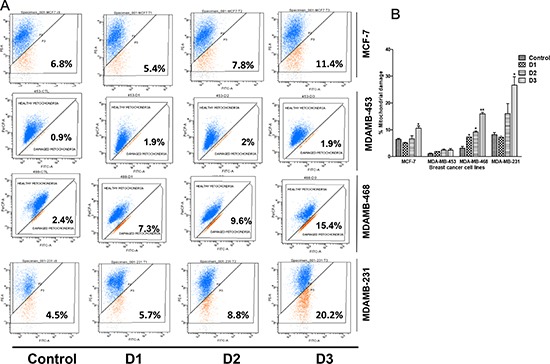
PP induces a loss of mitochondrial membrane potential Breast cancer cells were seeded in 6-well plates and treated with different concentrations of PP for 24 hr. Cells treated with the vehicle or with PP were stained with JC-1 and subjected to flow cytometric analysis. **(A)** Flow cytometric measurement of mitochondrial membrane potential **(B)** Histogram showing increased mitochondrial damage corresponds to decreased mitochondrial membrane potential. The average percentage (± SEM) of cells with decreased membrane potential is indicated. Asterisks indicate statistically significant differences between PP-treated and untreated cells, *p* < 0.05 (*), and *p* < 0.01(**), as determined by Student's t-test.

Previously, we showed that PP activates caspase-3 expression and concomitant cleavage of PARP [20]. Mitochondrial Δψm is a measure of the capacity of the respiratory chain to generate ATP. To determine if PP has an effect on Δψm, the cationic lipophilic dye, JC-1, which accumulates within the mitochondria in a potential-dependent manner, was used. Δψm was measured in all breast cancer cells by flow-cytometry (Figure 2A). PP treatment resulted in a concentration-dependent increase in mitochondrial damage (Figure 2B), which corresponded to decreased Δψm. This damage was more pronounced in MDA-MB-231 cells relative to other breast cancer cells. Thus, PP-mediated apoptosis in breast cancer cells is apparently through disruption of Δψm.

**Figure 3:**
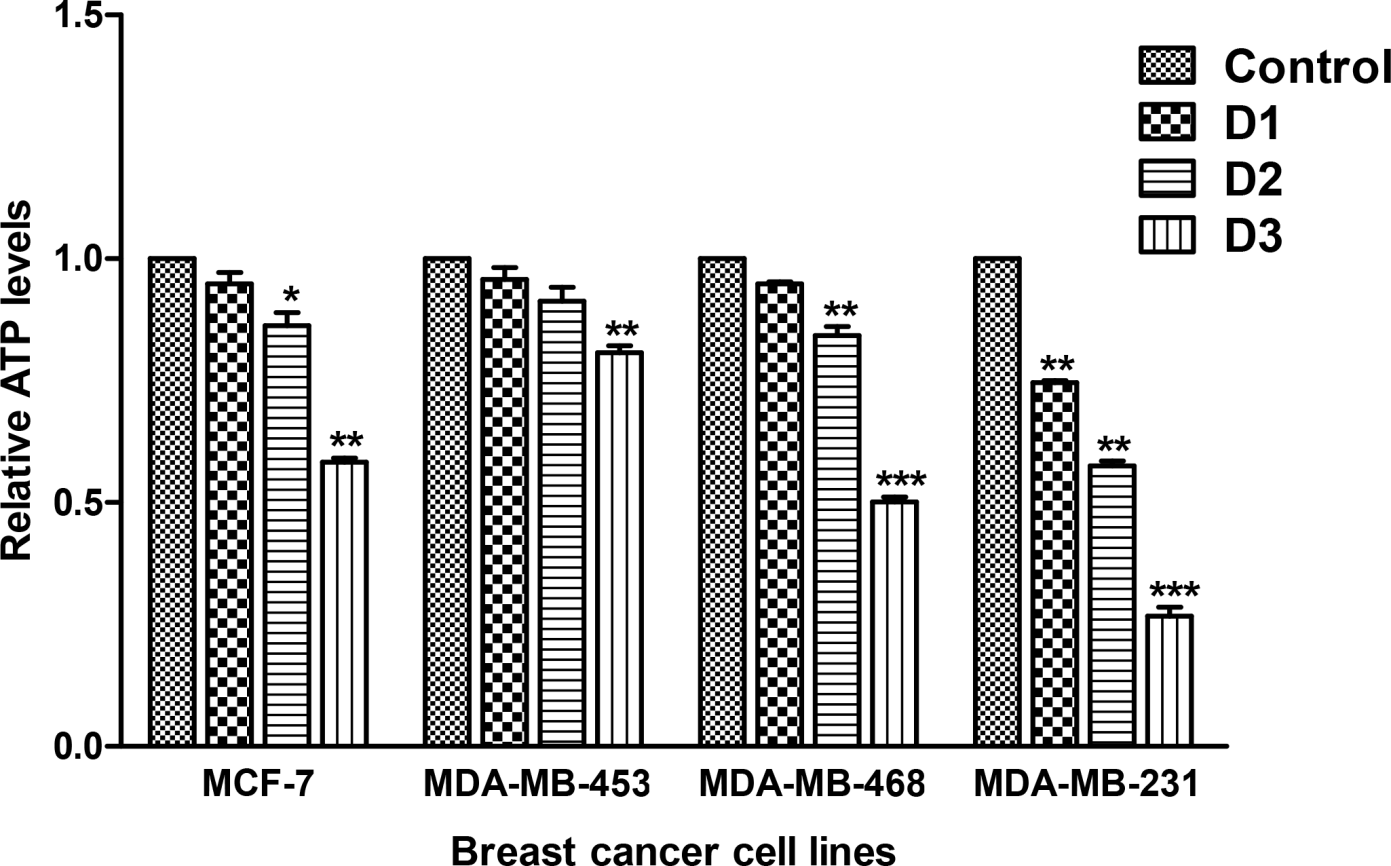
PP reduces ATP levels Breast cancer cells were treated with different concentrations of PP for 24 hr and counted. ATP levels (mean ± SEM, *n* = 3 experiments) were determined by a luciferin–luciferase-based assay on equal numbers of live cells. Asterisks indicate statistically significant differences between PP-treated and untreated cells, *p* < 0.05 (*), *p* < 0.01(**), and *p* < .001(***) as determined by Student's t-test.

Mitochondrial Δψm is necessary for the activity of ATP synthase, which generates ATP [23]. To determine the role of Δψm in ATP production, the effect of PP on ATP production was assessed. Decreases in ATP levels were observed in all PP-treated breast cancer cells relative to their respective controls (Figure 3). MDA-MB-453 cells were less sensitive than MCF-7 and the other TNBC cells.

### LDH-A and LDH-B expression

**Figure 4:**
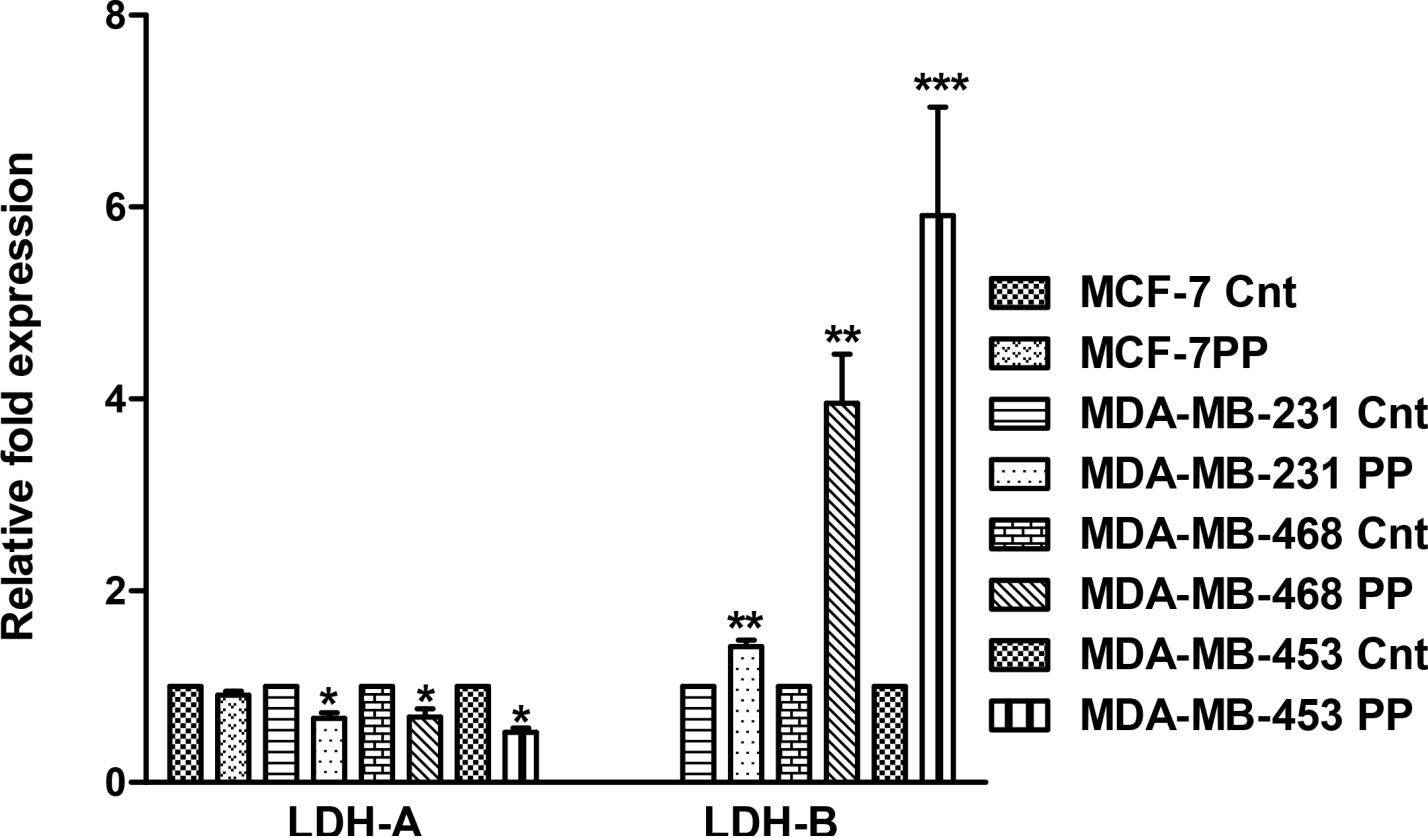
PP modulates expression of LDH-A and LDH-B qRT-PCR analysis of the mRNA expression of LDH-A and LDH-B was accomplished for breast cancer cells after PP treatment (D3 dose). Relative expressions of LDH-A and LDH-B as compared to respective controls are plotted in the graph. GAPDH was used as internal control. The data represented as mean ± standard deviation (*n* = 3). Asterisks indicate statistically significant differences between PP-treated and untreated cells, *p* < 0.05 (*), and *p* < 0.01(**), as determined by Student's t-test.

LDH-A and LDH-B expression levels in the breast cancer cell lines were determined by qRT-PCR. PP treatment caused a decrease in the LDH-A expression and increase in the LDH-B expression in all the cell lines. MCF-7 control and PP treated cells did not express LDH-B (Figure 4).

**Figure 5:**
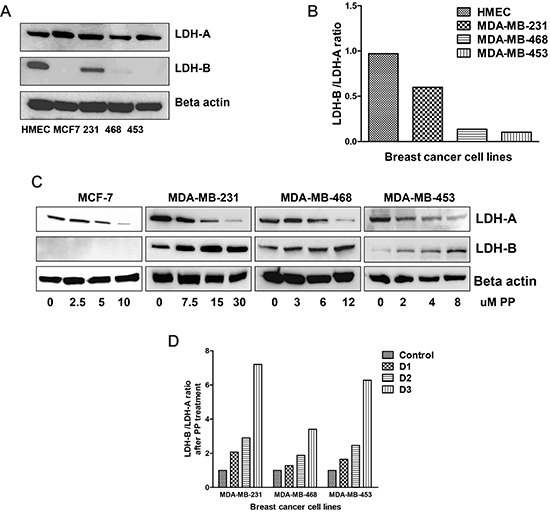
PP alters LDH protein expression Total protein was isolated from control and PP-treated breast cancer cells and subjected to immunoblotting. Membranes were stripped and re-probed with anti-actin antibody to ensure equal protein loading. **(A)** Immunoblotting of LDH-A and LDH-B in control cells. **(B)** Bar diagram indicating the ratio of LDH-B to LDH-A in control cells. **(C)** Immunoblotting of LDH-A and LDH-B in PP-treated cells. **(D)** Bar diagram indicating the ratio of LDH-B to LDH-A in PP-treated cells.

The basal expression levels of LDH-A and LDH-B proteins in all breast cancer cells and in normal human mammary epithelial cells (HMEC) were determined by immunoblotting. HMEC cells expressed both LDH-A and LDH-B at the protein level (Figure 5A). However, LDH-B expression was absent in MCF7. As previously reported [14], there was higher LDH-A expression in the breast cancer cells and almost similar level of expression was observed in HMEC cells; however, LDH-B expression was higher in HMEC cells relative to breast cancer cells (Figure 5A). The LDH-B expression was comparatively low in MDA-MB-453, and MDA-MB-468 and MDA-MB-231 cells.

PP treatment caused a decrease in ECAR, which corresponds to a decrease in lactate production through LDH-A. To confirm this, the expressions of LDH-A and LDH-B expression were determined in PP-treated breast cancer cells. There was up-regulation of LDH-B expression in these cells, except that, in MCF-7 cells, LDH-B expression was not observed (Figure 5B).

Since there was a loss of LDH-A and increased LDH-B expression after PP treatment, the ratio of LDH-B/LDH-A, i.e., the *Glycolytic Index* (GI), was calculated. A higher GI was observed for HMEC cells (0.9) relative to breast cancer cells (Figure 5C). PP treatment increased the LDH-B/LDH-A ratio in a concentration-dependent manner (Figure 5D). Thus, the GI is an indicator of utilization of OXPHOS instead of lactate by cancer cells for their energy needs, and thus provides a measurement for determining which cells exhibit the Warburg effect.

### Increased LDH-B reduces cell growth

**Figure 6:**
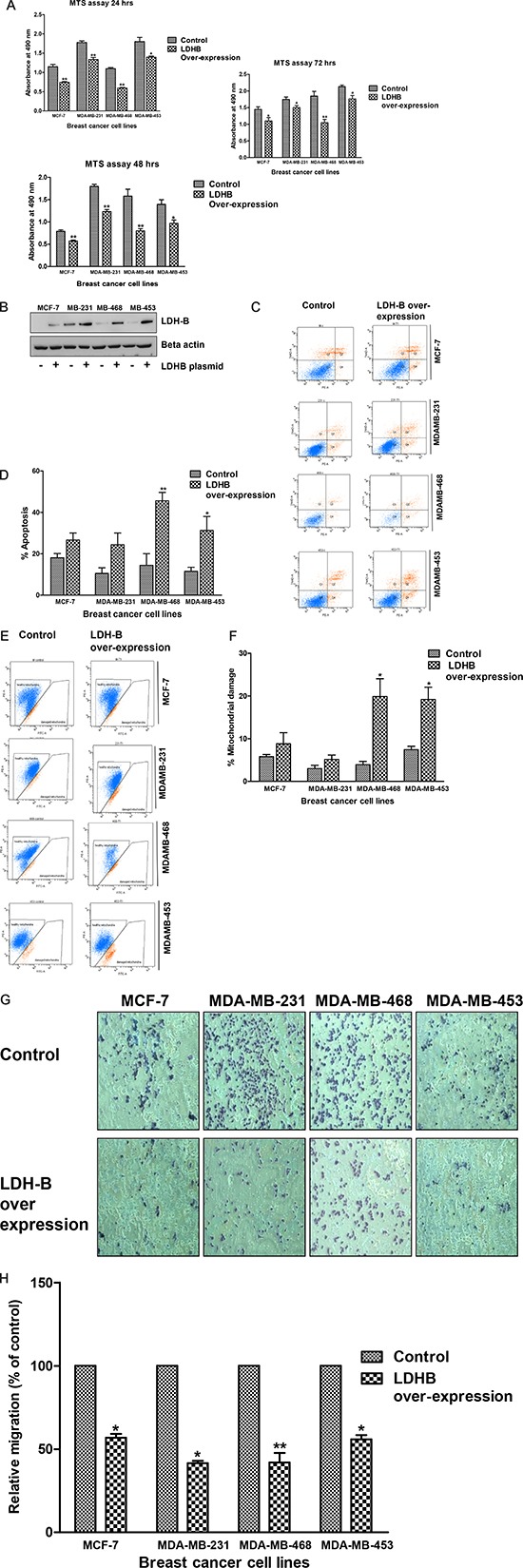
Over-expression of LDH-B leads to reduced viability, apoptosis and less mobility LDH-B was transiently over-expressed in MCF-7, MDA-MB-231, MDA-MB-468, and MBA-MD-453 cells. Effect of LDH-B over-expression was determined on cell viability assay, apoptosis, mitochondrial membrane potential, and migration assays. **(A)** Cell viability assay after ectopic LDH-B expression at 24, 48 and 72 hrs. **(B)** Immunoblotting of LDH-B to confirm its over-expression in breast cancer cells. **(C)** Flow cytometric measurement of apoptosis in breast cancer cells after LDH-B over-expression. **(D)** Histogram showing increased numbers of apoptotic cells in breast cancer cell lines after LDH-B over-expression. **(E)** Flow cytometric measurement of mitochondrial damage in breast cancer cells after LDH-B over-expression. **(F)** Histogram showing increased mitochondrial damage in breast cancer cell lines after LDH-B over-expression. **(G)** Microscopic photograph of cell migration after LDH-B over-expression. **(H)** Histogram showing decreased migration of breast cancer cell lines after LDH-B over-expression. The data represented as mean ± standard deviation (*n* = 3). Asterisks indicate statistically significant differences between PP-treated and untreated cells, *p* < 0.05 (*), and *p* < 0.01(**), as determined by Student's t-test.

Effect of ectopic LDH-B expression on breast cancer cell growth was determined at different time points. Compared with cells transfected with the scrambled plasmid DNA, cells transiently transfected with LDH-B plasmid DNA demonstrated lower viability (Figure 6A). The change in the viability over time was not detected in any of the cell line tested. These results suggested the role of LDH-B in the proliferation and survival of breast cancer cells.

### Over-expressed LDH-B induces apoptosis and reduces cell motility

To determine if high levels of LDH-B have an effect on apoptosis and cell motility, MCF-7, MDA-MB-231, MDA-MB-468, and MDA-MB-453 breast cancer cells were transiently transfected with an LDH-B plasmid (Figure 6B). Over-expression of LDH-B led to an increase in the percentage of apoptotic cells, as measured by PE Annexin apoptosis kits and analyzed by flow cytometry. MDA-MB-468 and MDA-MB-453 cells showed increases (2.7- to 3.2-fold) in apoptotic cells relative to their respective control cells (Figure 6C & 6D, *p* < 0.01 and *p* < 0.05 levels, respectively). LDH-B over-expression led to a 1.5–5 fold increase in the percentage of cells with damaged mitochondria, in particular, in MDA-MB-468 and MDA-MB-453 cells (Figure 6E & 6F, *p* < 0.05). Further, LDH-B over-expression in MCF-7, MDA-MB-231, MDA-MB-468, and MDA-MB-453 cells caused less cell migration relative to control (empty vector) cells. Their migration was reduced 2–3 fold (Figure 6G & 6H, *p* < 0.01 and *p* < 0.05).

**Figure 7:**
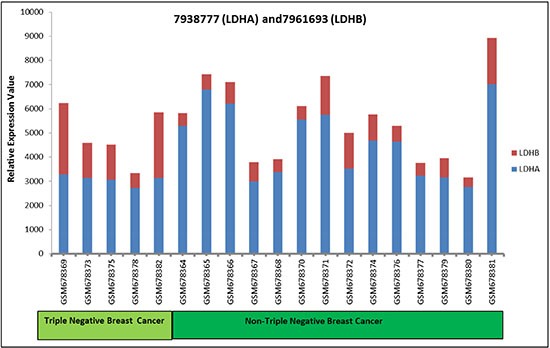
LDH-A and LDH-B expression in TNBC and non-TNBC breast cancer samples The Yang et al. data set was used to determine mRNA expression levels of LDH-A and LDH-B in breast tumor samples (5 TNBCs and 14 non-TNBCs). Data are based on a ratio of the raw signal intensities from the microarray GDS4069 NCBI/GEO database plotted on the y axis.

To determine the clinical significance of the LDH-B/LDH-A ratio, we analyzed individual mRNA expression levels of LDH-A and LDH-B using the Yang et al. GDS4069 data set available on the GEO website http://www.ncbi.nlm.nih.gov/geo/) [24]. Lower levels of LDH-B relative to LDH-A levels was observed in 14/14 non-TNBC breast cancers and in 3/5 TNBCs (Figure 7). Thus, LDH-B is generally lower in breast cancers. Further, the ratio of LDH-B to LDH-A would be more useful than either of these markers alone.

## DISCUSSION

A distinguishing feature of cancer cells relative to normal cells is bioenergetics. Lactate provides cancer cells with a major source of energy; a phenomenon known as the Warburg effect. In contrast, normal cells rely mainly on oxidative phosphorylation [2, 25]. Targeting metabolism is a new approach for treatment of cancer, especially to overcome therapeutic resistance [26]. Currently, there is a focus on inhibition of enzymes involved in energy metabolism. LDH-A has gained attention, as it is up-regulated in many tumors and is involved in tumor initiation and growth [27]. In contrast, there have been contradictory reports on the role of LDH-B in breast tumors [17, 19, 28].

Our results indicate that both LDH-A and LDH-B have functions in breast cancer and that their levels can be modulated through a pharmacological inhibitor, PP, which targets NF-κB. The results show that PP reduces Δψm and ATP levels in breast cancer cells. Previous studies also reported decreased ATP level in the mitochondria-targeted vitamin E analog (Mito-chromanol, Mito-ChM) and mitochondrial ErBB2 over-expressing cells. Decreased ATP level in MCF-7 and MDA-MB-231 cells identified to be effective in inhibiting energy metabolism in breast cancer cells and in mice xenografts [29, 30]. Thus, PP induces nonproductive mitochondrial respiration, as reported previously for cells with knockout LDH-A and for cells treated with FX-11 (LDH-A inhibitor) [14, 27].

Our observed changes in the LDH-A, LDH-B level correlates with the changes in the OCR and ECAR. Reduced LDH-A reflect decrease in lactate production as we also observed decreased ECAR level. We detected increased LDH-B level after PP treatment similarly we find increase in the OCR to ECAR ratio which indicates total OXPHOS. An abrogated LDH-A and induced LDH-B expression both are capable of generating more pyruvate for further utilization in OXPHOS.

Although LDH-A was over-expressed in all the breast cancer cell lines examined, expression of LDH-B was higher in HMEC cells relative to breast cancer cells. In MCF-7 cells, LDH-B expression was not found, an observation similar to previous reports, with one exception [31]. Thus we were unable to access the function of LDH-B in this cell line.

The LDH-B promoter is hypermethylated in breast and prostate cancers [17, 18], and PP treatment results in down-regulation of LDH-A in four different breast cancer cell lines and up-regulation LDH-B in three cell lines. This suggests the possibility that PP, via modulation of NF-κB, is involved in demethylation of the LDH-B promoter. This premise is supported by our previous work showing that PP treatment results in increased expression of E-cadherin, the promoter of which is hypermethylated in MDA-MB-231 breast cancer cells [22, 32].

**Figure 8:**
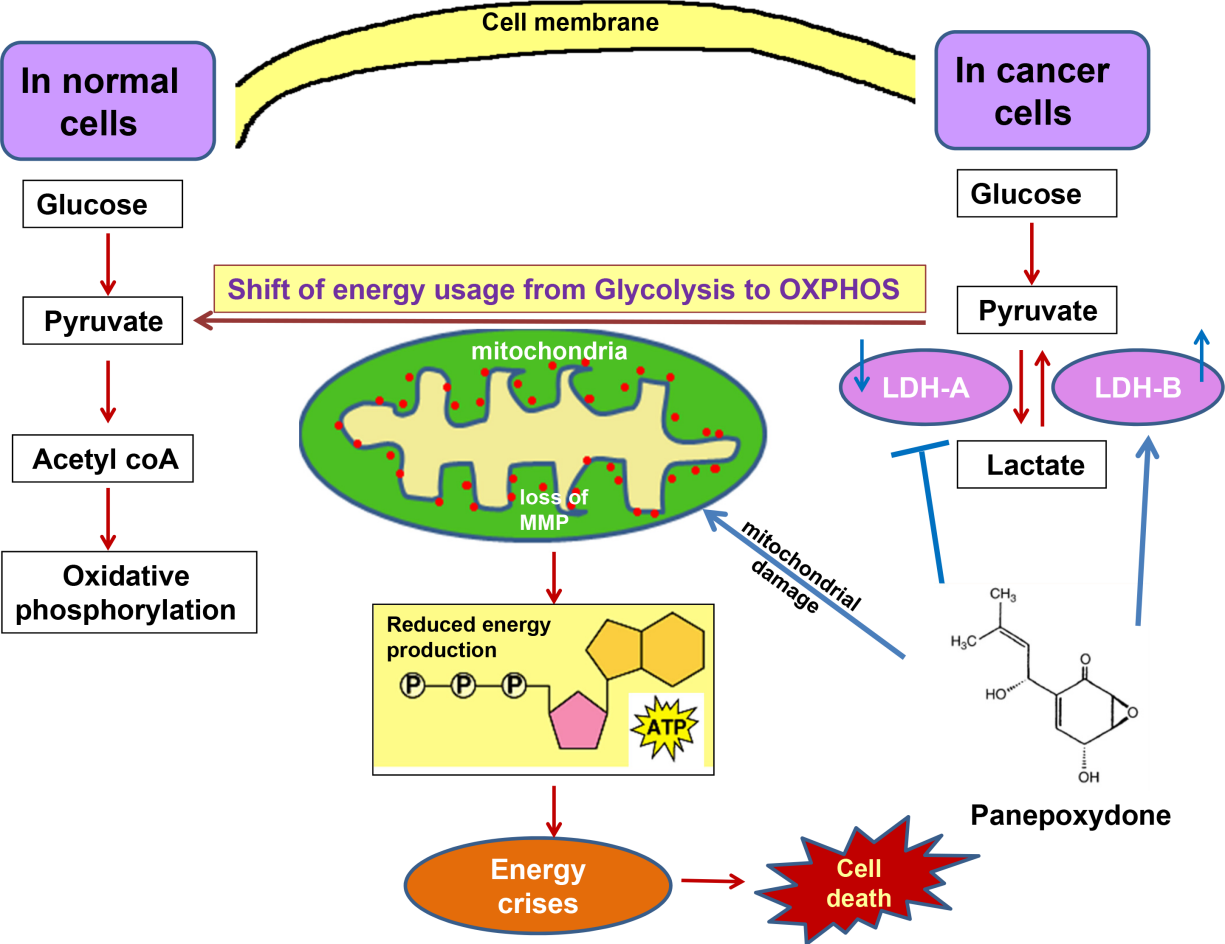
A proposed model of PP targeted metabolic alterations PP down-regulates LDH-A and up-regulates LDH-B to generate more pyruvate, which is available for OXPHOS. The shift in energy usage may change the phenotype of cells. PP also reduces mitochondrial Δψm, which leads to energy crises and to cell death.

There was also a decrease in Δψm after PP treatment and after over-expression of LDH-B. Since LDH-A is responsible for converting pyruvate to lactate, its restriction should lead to an accumulation of pyruvate, thus making pyruvate available as a source to generate ATP for the metabolic demands of cell growth [33, 34]. Once the OXPHOS chain is activated, more electrons are generated from the chain and, after combining with oxygen, form reactive oxygen species, which damage the mitochondrial membrane and cause mitochondrial pathway apoptosis [35]. Thus, decreased Δψm supports a role for the mitochondrial pathway during apoptosis. In summary, our data support the hypothesis that accumulation of pyruvate through LDH-A inhibition and/or re-expression of LDH-B is required for cancer cells to utilize OXPHOS as their energy source (Figure 8).

Interestingly, ectopic expression of LDH-B has significantly decreased the Δψm and induced apoptosis in MDA-MB-468 and MDA-MB-453 cells but in MCF-7 and MDA-MB-231 cells the changes were not significant. The reason for this could be because of the differential behavior of the TNBC cells. Each TNBC behave differently and that is why we need different treatment therapy for specific tumor type.

LDH-A is a biomarker for glycolysis activity. Further, its expression positively correlates with tumor size, indicating that LDH-A expression influences tumor cell proliferation and that inhibition of LDH-A augments apoptosis [36]. As shown here, LDH-B over-expression resulted in an increase in apoptosis in all breast cancer cell lines, even the LDH-B deficient MCF-7 cells. The induction of apoptosis is one of the protective mechanisms against cancer initiation and progression [37]. Our findings are similar to those related to inhibition of glycolysis, for which specific inhibitors, 3-bromopyruvate and 2-deoxyglucose, result in mitochondrial pathway-induced apoptosis [38, 39]. Thus, targeting NF-κB with pharmacological inhibitors, such as PP, could induce mitochondrial pathway apoptosis through damage to the mitochondrial membrane. More work should be accomplished to determine the clinical relevance of targeting NF-κB for breast cancer patients.

It is well documented that the intracellular ratio of Bax/Bcl-2 protein can strongly influence the ability of a cell to respond to an apoptotic signal [40]. Similarly, our results indicate that the LDH-B/LDH-A ratio provides a way to identify tumor cells with aggressive behavior. High levels of LDH-B are present in both benign and non-malignant prostate and breast tissue [17, 18]. The fact that PP, which causes a reversal of aggressive features, also causes increased expression of LDH-B in breast cancer cells, similar to HMECs, suggests that LDH-B generates more pyruvate, a substrate for the tricarboxylic acid cycle, followed by OXPHOS, thus rendering the cells less dependent on lactate for cellular respiration.

Altogether, our findings suggest that the ratio of LDH-B/LDH-A is more relevant than either LDH-A or LDH-B expression alone. LDH-B expression is up-regulated in basal-like TNBCs [28]. Our analysis of 5 TNBCs and 14 non-TNBCs support the concept that LDH-B levels are higher in TNBCs. Nevertheless, LDH-B was lower than LDH-A in 3/5 TNBCs. Further, a lower LDH-B/LDH-A ratio was observed in 100% of non-TNBCs. Thus, the LDH-B/LDH-A ratio could be a biomarker of tumor aggressiveness, particularly as it relates to breast cancer subtypes in TNBCs, particularly the luminal versus basal-like subtypes, which have different prognoses [41]. Targeting of both LDH-A and LDH-B could be a promising therapeutic strategy for the treatment of breast cancer.

The present study has a limitation as it does not a include a large patient cohort to validate the LDH-A and LDH-B expression level, however it does highlight the possibly that the LDH-B/LDH-A ratio could be utilized to determine the glycolytic index of breast tumors. Non-invasive assays, which accurately reflect the “state of the tumor” are essential to not only detect tumors earlier, but to monitor treatment effectiveness as well. Since LDH is already utilized in the clinic for other cancer types, it is possible that the LDH-B/LDH-A ratio could be better or more effective method to further characterize breast tumors, however judicious selection of which breast cancer subtypes is essential to accurately use this index to monitor treatment response. Furthermore, since increased glycolysis leads to chemoresistance in breast cancer, determining the LDH-B/LDH-A ratio, would suggest a rationale that either NF-kB or LDH-A inhibitors could be added to current treatment regimens.

## MATERIALS AND METHODS

### Drug and reagents

Panepoxydone (PP), a natural NF-ĸB inhibitor, was purchased from Alexis Biochemicals (San Diego, CA). Dimethyl sulfoxide (DMSO, vehicle control), Triton X-100, and bovine serum albumin (BSA) were obtained from Sigma-Aldrich (St. Louis, MO). Dulbecco's Modified Eagle Medium (DMEM), fetal bovine serum (FBS), trypsin-EDTA, penicillin, and streptomycin were purchased from Invitrogen (Carlsbad, CA). For measurement of ATP, the CellTiter-Glo Assay was purchased from Promega (Madison, WI) and JC-1 dye from Sigma-Aldrich (St. Louis, MO). Primary antibodies against LDH-A and LDH-B were purchased from Cell Signaling Technology (Beverly, MA) and Abcam (Cambridge, MA), respectively. Anti-rabbit and horseradish peroxidase-conjugated secondary antibodies were obtained from Santa Cruz Biotechnology (Santa Cruz, CA). The β-actin (mouse monoclonal) antibody was from Sigma-Aldrich (St. Louis, MO).

### Human breast cancer cell lines and culture conditions

The estrogen receptor-positive MCF-7 cell line and three TNBC cell lines (MDA-MB-231, MDA-MB-468, and MDA-MB-453) were acquired from ATCC (Manassas, VA) and cultured in DMEM supplemented with 10% fetal bovine serum, penicillin (100 U/ml), and streptomycin (100 μg/ml) in a humidified 5% CO_2_ incubator at 37°C. Cells were sub-cultured biweekly with a split ratio of 1:3. For treatments, a stock solution of PP (50 mM) was prepared in DMSO and stored at −20°C in aliquots. At the time of experiments, dilutions were freshly prepared in complete growth medium. Equal volumes of DMSO (final concentration, 0.2%) were added to the controls.

### Cell metabolism assays

Rates of glycolysis and oxidative phosphorylation were determined by measuring the oxygen consumption rates (OCR) and extracellular acidification rates (ECAR, a measure of lactic acid release) by use of a Seahorse XF24 Analyzer (Seahorse Bioscience, Billerica, MA). Briefly, 5 × 10^4^ cells/well were seeded in XF24 cell culture microplates (Seahorse Bioscience). After 4–5 hr, cells were treated with different concentrations of PP (based on IC_50_ values), and plates were incubated at 37°C for 24 hr. The next day, medium was changed to XF Assay Media, and the plates were loaded into the XF24 analyzer. Data were collected and analyzed.

### Mitochondrial membrane potential measurement

The lipophilic cationic dye JC-1 (5,5′,6, 6′-tetrachloro-1,1′,3,3′tetraethyl- benzimidazolcarbocyanine iodide) was used to detect Δψm of the cell lines. 3 × 10^5^ cells were seeded on 6-well plates and, after overnight incubation, treated with PP for 24 hr. Cells were collected and stained with JC-1 (10 μg/ml) at 37°C for 30 min. Formation of J-aggregates was assessed by flow cytometry.

### ATP analysis

Cellular ATP content was determined by a luciferin–luciferase-based bioluminescence assay (Promega, Madison, WI), as outlined in the manufacturer's protocol. MCF-7, MDA-MB-231, MDA-MB-468, and MDA-MB-453 cells were treated with PP for 24 hr and counted. ATP levels were determined on equal numbers of cells (5000/well) according to a standard protocol. Equal volumes of CellTiter-Glo reagent were added and mixed for 2 min, followed by 10 min of incubation at room temperature (RT). Luminescence was measured using a VictorV (PerkinElmer, Waltham, MA) plate reader.

### cDNA synthesis and *Real-time RT-PCR*

Total RNA was extracted from control and high dose PP-treated cells (double IC_50_ value) with Trizol reagent (Sigma-Aldrich, Dorset, UK) according to the manufacturer's instructions. RNA was quantified spectrophotometrically. RNA (2 μg) was reverse-transcribed into cDNA using a High Capacity cDNA Reverse Transcription Kit (Applied Biosystems, Carlsbad, CA) with 250 ng of random primers according to the manufacturer's instructions.

Quantitative real-time PCR was performed in 96-well plates using SYBR Green Master Mix (Roche) on an iCycler system (Bio-Rad, Hercules, CA) with previously published primers [42]. The thermal conditions for real-time PCR assays were as follows: cycle 1: 95°C for 10 min, cycle 2 (x40): 95°C for 10 sec and 58°C for 45 sec. Threshold cycle (CT) values for LDH-A and LDH-B were normalized against CT values for control GAPDH, and a relative fold-change in expression with respect to a control sample was calculated by the 2^−ΔΔCt^ method.

### Immunoblotting

Cells exposed to various concentrations of PP for 24 hr were lysed, and protein concentrations were determined with DC Protein Assay kits (Biorad, Hercules, CA) following the manufacturer's instructions. Total protein (80 μg) of each cell lysate were subjected to resolution on 10% sodium dodecyl sulfate polyacrylamide gel electrophoresis (SDS-PAGE) and electro-transferred onto polyvinylidene difluoride (PVDF) membranes. The membranes were incubated with blocking buffer (5% non-fat dry milk in phosphate-buffered saline) for 1 hr at room temperature (RT) and then incubated with specific antibodies diluted 1:1000 times in 5% non-fat dry milk overnight at 4°C. After washing with a Tris-buffered saline solution containing 0.1% Tween 20 (TBST), membranes were incubated with horseradish peroxidase-conjugated secondary antibodies for 2 hr at RT followed by washing with TBST. Blots were then treated with chemiluminescence reagents using Super Signal West Femto Kits (Pierce, Rockford, IL), and signals were detected with an LAS-3000 image analyzer (Fuji Photo Film Co., Tokyo, Japan). Each membrane was stripped and re-probed with anti-β-actin antibody to ensure equal protein loading. Densitometry was performed with an AlphaImager (Alpha Innotech Corp., San Leandro, CA).

### Transient transfection for over-expression of LDH-B

The LDH-B plasmid (pCMV-6-AC-GFP vector) was expanded by use of One Shot^®^ TOP10 chemically competent *E. coli* cells (Invitrogen) following the standard transformation procedure on LB agar plates supplemented with ampicillin [43]. Plasmids were isolated, and MCF-7, MDA-MB-231, MDA-MB-468, and MDA-MB-453 cells were transiently transfected. Briefly, 1 × 10^5^ cells were seeded in 6-well plates. After overnight incubation, cells were transfected with the LDH-B expression plasmid (2 μg) or pCMV-6-AC-GFP control plasmid using Lipofectamine RNAiMax (Invitrogen) as a transfection reagent. After overnight incubation, media containing the transfection mixture was replaced with fresh serum-containing media and incubated for 48 hr. Cells collected after 48 hr of transfection were utilized for cell migration, apoptosis, mitochondrial Δψm assays, and Western blot analyses. Cell migration and apoptosis procedures were followed as reported previously [20].

### Cell viability assay

The effect of ectopic expression of LDH-B on cell growth was measured using AQueous One Solution Cell Proliferation Assay (MTS assay, Promega) according to the manufacturer's protocol. Briefly, MCF-7, MDA-MB-231, MDA-MB-468 and MDA-MB-453 cells seeded in 96-well plates (5,000 cells/well) and were transfected with control and plasmid DNA as described above. After 24, 48 and 72 hrs of transfection, medium was removed and 20 ul MTS reagent with 100 μl media was added to each well. Plates were incubated at 37°C for 30 min-2 hrs. The fluorescent intensity was measured at 490 nm using Gen5 (Beckman coulter, Inc., Brea, CA, USA).

### Statistical analysis

Student's t-test was used to evaluate the statistical significance of the results. *p* < 0.05 was considered statistically significant. All the data analysis was done using GraphPad Prism version 5.0 software and graphs were also created using this software.

## SUPPLEMENTARY METHOD AND FIGURE


