# Supplementary material for: Inhibition of the Warburg effect with a natural compound reveals a novel measurement for determining the metastatic potential of breast cancers
Source: Oncotarget. 2015 Jan 9;6(2):662–78. doi: 10.18632/oncotarget.2689 (PMC4359247; doi:10.18632/oncotarget.2689)
Supplement: Supplementary file 1 [file oncotarget-06-662-s001.pdf]

SUPPLEMENTARY METHOD AND FIGURE

In vitro colony survival assay

MDA-MB-231, MCF-7,MDA-MB-453,MDA-MB-468 cells plated in 96-well plate at the density of ~100 cells per well and next day treated with appropriate IC_{50} concentrations of Panepoxydone for 48 hrs, washed and replaced with regular DMEM-medium. A colony was arbitrarily defined to consist of at least 50 cells. After 15 days, colonies were fixed with 4% formaldehyde, stained with crystal violet and counted.

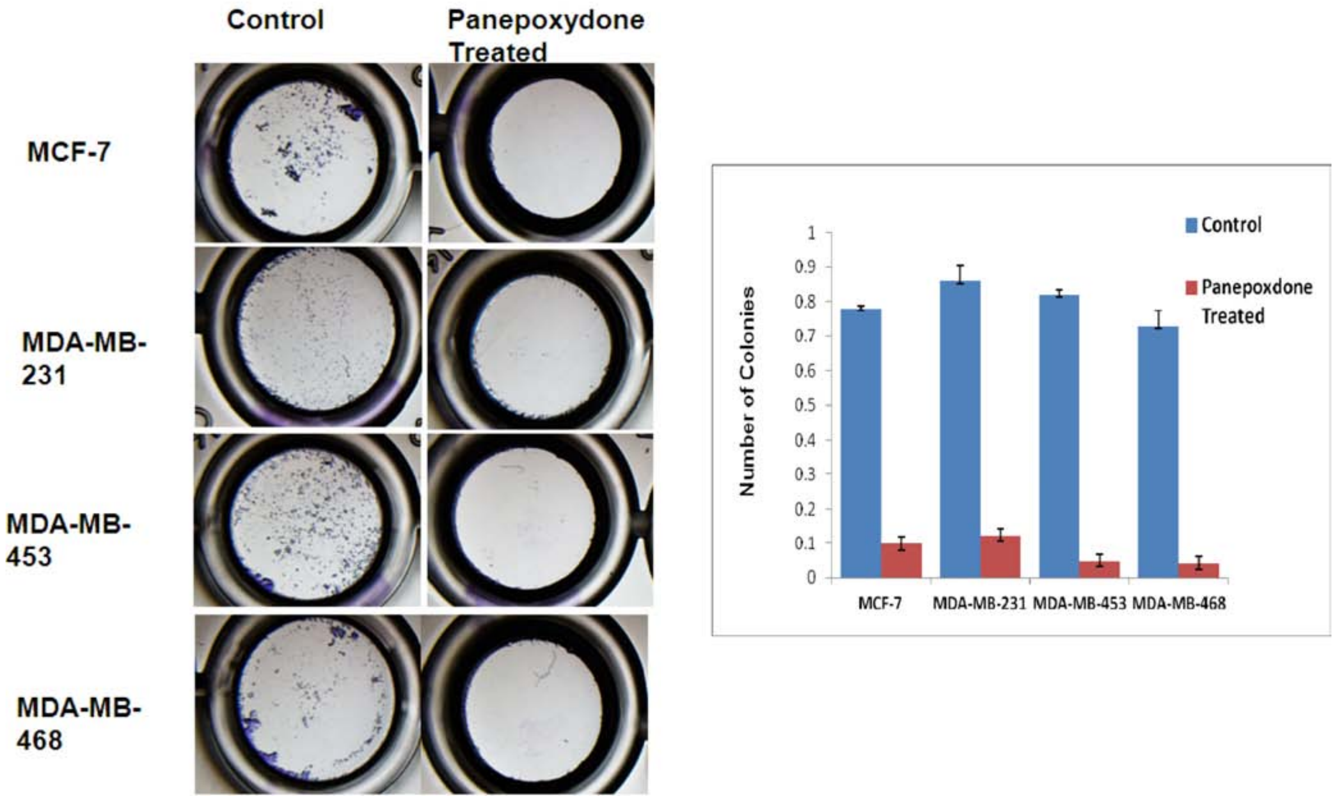

Supplementary Figure S1: PP reduces colony formation in breast cancer cells. MCF-7, MDA-MB-231, MDA-MB-468, and MDA-MB-453 cells were plated (100 cells) in 96 well plates. Bar-graph representation and photograph of crystal violet-stained surviving colonies from control and Panepoxydone-treated groups are shown. After 15 days colonies were stained with crystal violet and counted. Bar graph created on average of three values.
